# Postoperative Ileus: Comparative Pathophysiology and Future Therapies

**DOI:** 10.3389/fvets.2021.714800

**Published:** 2021-09-13

**Authors:** Emily A. Hellstrom, Amanda L. Ziegler, Anthony T. Blikslager

**Affiliations:** Department of Clinical Sciences, North Carolina State University, Raleigh, NC, United States

**Keywords:** intestine, equine, surgery, barrier function, enteric glia and neurons, microbiota

## Abstract

Postoperative ileus (POI), a decrease in gastrointestinal motility after surgery, is an important problem facing human and veterinary patients. 37.5% of horses that develop POI following small intestinal (SI) resection will not survive to discharge. The two major components of POI pathophysiology are a neurogenic phase which is then propagated by an inflammatory phase. Perioperative care has been implicated, namely the use of opioid therapy, inappropriate fluid therapy and electrolyte imbalances. Current therapy for POI variably includes an early return to feeding to induce physiological motility, reducing the inflammatory response with agents such as non-steroidal anti-inflammatory drugs (NSAIDs), and use of prokinetic therapy such as lidocaine. However, optimal management of POI remains controversial. Further understanding of the roles of the gastrointestinal microbiota, intestinal barrier function, the post-surgical inflammatory response, as well as enteric glial cells, a component of the enteric nervous system, in modulating postoperative gastrointestinal motility and the pathogenesis of POI may provide future targets for prevention and/or therapy of POI.

## Introduction

Postoperative ileus (POI), “a delay in the return of normal gastrointestinal motility following surgery” is a disease syndrome that has become increasingly important to manage in both veterinary and human health (1, 2). POI is a complex syndrome that first requires healthcare professionals to agree on correct terminology and definition in order to properly recognize and treat it. Among human healthcare professionals, the American Society for Enhanced Recovery After Surgery (ERAS) has defined a scoring system for what they termed “postoperative gastrointestinal dysfunction” (POGD) (3, 4). The system describes varying degrees of POI with a 0–2 normal score as nausea and vomiting within the first 24–48 h and progresses all the way to scores over 6 that qualify as POGD with painful abdominal distension with tympany, unrelenting nausea when treated with antiemetics, no bowel movements, and large volumes of bilious emesis (3, 4). In the equine field, efforts have been made to define POI as exhibiting over 4 L of gastric reflux with a pH >4.0 upon nasogastric intubation or 2 L/h with repeated intubation, persistent heart rate over 40 beats/min, abdominal discomfort, and evidence of distended SI on ultrasound or rectal palpation (2, 5). Due to variations in past terminology, the ranges of reported incidence rates are wide. Incidence is thought to be between 2 and 60% in human patients, and equine incidence following abdominal surgery is thought to be between 0 and 62% (2, 6–8). Even with a wide range of reported incidence, POI is estimated to cost the US healthcare 1.5 billion dollars each year, and it is well established that increased time within a hospital increases complications (8–13). POI is a critical postoperative complication in the equine field. Horses that develop POI following SI resection are 29.7 times less likely to survive to discharge than similar patients without POI (14). A unique aspect of equine veterinary medicine is that acute abdominal pain, or more commonly referred to as colic, is the most common medical emergency (15). Up to 17.5% of colicking horses require exploratory laparotomy (16–23). With this high incidence of equine patients undergoing gastrointestinal surgery and the significant impact of POI on patient survival rate, it is clear that the equine veterinary field provides a significant opportunity for the study of POI pathophysiology and therapy that will aid human health. In order to properly treat and prevent POI, an attempt must be made to understand its multifactorial pathophysiology. Combining the current knowledge that POI is incited by a rapid neurogenic response and then propagated through an inflammatory response with a better understanding of how breakdown of the intestinal mucosal barrier resulting in translocation of luminal contents contributes to the inflammatory response, and an increasing interest of the role of the microbiota and enteric glia in physiologic function of the gastrointestinal tract, we may hope to develop more successful interventions for our patients.

## RISK FACTORS

While POI is a multifactorial disease process, medical professionals have been able to observe a variety of risk factors in both human and equine patients. Both species share a correlation between increased age and increased risk of POI as well as hypovolemia (2, 9–13, 24). In human patients, hypovolemia is noted specifically as blood loss in surgery, while in equine patients it is described as status at admission with tachycardia, increased packed cell volume, and increased serum total protein (2, 9–13, 24). Human risk factors for POI also include male gender, history of airway disease, and perioperative opiate use. Equine patients with SI surgical lesions are known to have an increased risk of POI, while human patients undergoing an ileostomy procedure are also at increased risk (2, 9–13, 24).

## Neurogenic Pathophysiology

**Figure 1:**
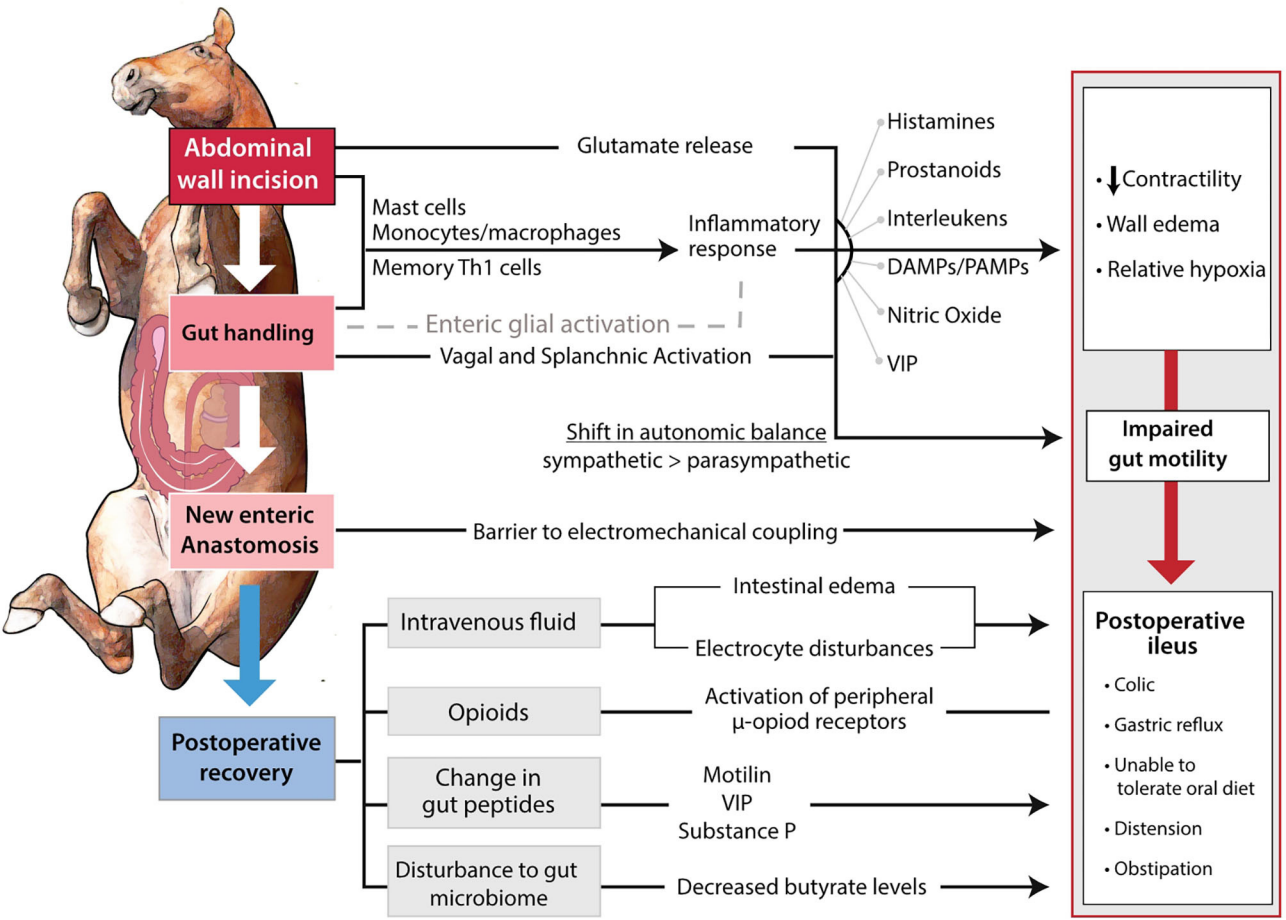
Pathways involved in the development of POI. The surgical incision and manipulation of tissue incite a neurogenic and inflammatory response. The combination of shift in autonomic balance and inflammatory products impair gastrointestinal motility by edema formation and decreased contractility. The impaired gastrointestinal motility is further propagated through the anastomosis site serving as an electromechanical barrier. Postoperatively, improper fluid therapy, opioid use, and potential changes to gastrointestinal peptides, further suppress motility.

The development of POI is first incited by a neurogenic response to surgery. Figure 1 demonstrates an outline of how the progression of a surgical procedure leads to dysmotility (24). There is an overall shift in the relative activity of the two major branches of the autonomic nervous system, from normal parasympathetic tone that serves to regulate physiological motility patterns, to an abnormal level of sympathetic tone that tends to reduce motility, potentially leading to functional obstruction (24). Although it would be intuitive to conclude that POI is initiated at the intestinal level, in actuality, POI pathophysiology is initiated with an abdominal incision causing a glutamate release and an adrenergic inhibition of motility (25–28). A study in which a non-selective adrenoceptor antagonist, guanethidine, or an alpha two adrenoceptor antagonist, yohimbine, improved colon motility postoperatively in rats supports this notion (29). The trauma of intestinal manipulation is sensed by the vagus afferent nerves which signal to the hypothalamus (2). The hypothalamus activates a shift toward sympathetic neuronal control, and motility is further inhibited (2). There is also thought to be activation of splanchnic afferent neurons that leads to a non-adrenergic and non-cholinergic stimulation of the vagus nerve and reduction of gastrointestinal motility by vasoactive intestinal peptide and nitric oxide (25, 30). This pathway was demonstrated by adrenergic blockade only partially preventing decreased intestinal motility in rats. The addition of nitrergic blockade to adrenergic blockade successfully prevented POI in this model (25, 31). The impact of vagal and splanchnic activation on reduced gastrointestinal motility from abdominal surgery has also been demonstrated by an observed improvement to motility by cutting both of the nerves in dogs (32). Similarly, gastrointestinal motility following surgery has also been improved in human patients by high epidural local anesthesia (33–36). Most of the neuronal effect is thought to be abated after surgery is completed, however, it is important to consider the effects of a resection and anastomosis procedure on coordination of peristaltic waves (Figure 1) (37, 38). A study performed in mice receiving a SI resection demonstrated an impact on the interstitial cells of Cajal, and ultimately, slow waves and contractions (23). Similar effects have been observed in human distal colon resections (39, 40).

## Inflammatory Pathophysiology

The decreased motility induced by the initial neurogenic response is further compounded through an inflammatory response in the intestinal muscularis (Figure 1). The peritoneal incision and intestinal manipulation induce the release of proinflammatory molecules in a complex process that requires further definition (41, 42). It has been theorized that tissue handling increases epithelial permeability, which allows pathogen-associated molecular patterns (PAMPs) to travel into the tissue from the intestinal lumen (43). This underexplored mechanism may provide a method of POI prevention to be shown in further detail later. Further, it is hypothesized that the resident muscularis macrophages are stimulated and release prostaglandins and nitric oxide (44, 45). It is important to note that while murine macrophages have demonstrated release of nitric oxide *in vitro*, this has yet to be show in human and equine macrophages (46, 47). Monocytes, mast cells, and neutrophils enter the muscularis and the leukocytes release inflammatory molecules including inflammatory cytokines, reactive oxygen intermediates, and protease (44, 48–52). Leukocytic chemotaxis has been noted at 3 h postoperatively and is thought to peak at 24 h postoperatively (2). Infiltration of the intestine by inflammatory cells is thought to impair motility by inducing intestinal wall edema via increased capillary permeability and the relaxing effect of nitric oxide and prostaglandin E2 on smooth muscle (53–56). Both the innate and the adaptive immune system are thought to be involved in this inflammatory response. A study by Engel et al. (57) utilizing mice provided a major shift in our understanding of POI by suggesting that the spread of dysmotility throughout the gastrointestinal tract was through the involvement of memory Th1 cells and their activation by local dendritic cells. The impact of inflammation on development of POI has been demonstrated by the administration of anti-inflammatory IL-10 improving motility postoperatively in mice (2, 58). Murine studies have also demonstrated the prevention of POI through inhibiting mast cell and leukocyte chemotaxis (49–52). Interestingly, the prevention of monocyte chemotaxis was not successful in preventing POI and resulted in more neutrophil infiltration into the muscularis externa (59). Other experimental models in mice have shown the improvement of POI in response to mast cell stabilization, macrophage depletion *via* chlodronate liposomes, or electric vagal stimulation to impair macrophage activation (50, 52, 60, 61). Mast cell stabilization was not successful in improving POI in humans, but laparoscopic surgery has been shown to decrease their activation (51, 62). Additional factors such as the need to exteriorize the intestine, decompress its contents, or perform a resection and anastomosis will increase the inflammation within the intestinal muscularis (63, 64).

An interesting aspect of POI is that while only certain sections of the gastrointestinal tract may be handled during surgery, ileus is commonly panenteric. As previously mentioned in the inflammatory response, activation of memory Th1 cells by IL-12 from dendritic cells at the location of manipulation could contribute to the panenteric decrease in motility by their traveling through the bloodstream (57). It has been speculated that the movement of commensal endotoxins, and PAMPs into the intestinal muscularis due to increased permeability from tissue handling can cause systemic inflammation leading to reduced motility (65). Panenteric decreased motility has also been thought to be attributed to mast cell mediators spread throughout the peritoneum (49, 54, 66). Opioids and electrolyte imbalances would additionally have systemic effects (24).

The pivotal study by Engel et al. (57) hypothesized that intestinal manipulation leads to dendritic cells releasing IL-12 which brings memory Th1 cells to the gastrointestinal tract where they simulate resident macrophages with interferon γ, and the macrophages release nitric oxide. They were able to support the role of dendritic cells and Th1 cells by first showing in a co-culture *in vitro* model that Th1 cells would release interferon γ and second by conditional knockout of CD11C [+] dendritic cells in mice. They observed that the conditional knockout mice did not develop POI after intestinal manipulation. They also demonstrated that interferon γ knockout mice had improved motility when compared to control, and IL-12 p35 knockout mice had normal motility. These results illustrate the need for further study of the role of intestinal manipulation in POI pathophysiology.

## Additional Pathophysiology Components

Neuropeptides, electrolytes, fluid therapy, and opioid use are also thought to contribute to POI development. As previously discussed, the inhibitory neuropeptide vasoactive intestinal peptide is thought to be involved in the non-adrenergic and non-cholinergic stimulation of the vagus nerve (25, 30). Surgery and postoperative fasting impact the levels of vasoactive intestinal peptide, and other stimulatory neuropeptides such as motilin and substance P (24). However, an understanding of neuropeptide levels in patients is required before potential new therapeutic targets may be developed (24). Electrolyte imbalances including hypokalemia, hypocalcemia, and hyponatremia have been observed to be significantly associated with POI and should be corrected in affected patients (9, 24). Anesthetic drugs are known to inhibit gastrointestinal motility, however, their use is unavoidable in many procedures. Opioids functioning at the u-opioid receptor inhibit gut motility through inhibition of acetylcholine release which in turn increases smooth muscle tone (2, 67). Its involvement in human POI is supported by the ability of alvimopan, a μ-opioid receptor antagonist, to increase postoperative recovery (68). This knowledge of opioid involvement appears to go underappreciated in the equine field, where a survey found that “87% of those reporting a POI incidence greater than the median incidence, declared the use of opioids in their treatment regiments” (2, 69, 70). Inappropriate fluid therapy may contribute to POI as a result of intestinal wall edema mentioned previously with inflammation, or electrolyte imbalance (24). Studies in human patients have demonstrated an association between ileus and crystalloid overload and report that for each additional liter of fluid received perioperatively, the overall risk of postoperative complications increases by 32% (71, 72).

## Current Therapies

The current therapies for postoperative ileus are broad to address the multifactorial pathology, but unfortunately, are still largely ineffective. For example, NSAID therapy has been utilized to target the role of the inflammatory response in POI development. NSAID therapy has been shown to decrease time to first flatus and stool in human patients undergoing colorectal surgery (73). However, it is imperative to note that the negative impact of nonselective COX inhibition on intestinal healing continues to make it a controversial postoperative therapy (73). In addition, in a study performed on the effects of nonselective COX inhibition and COX-2 selective inhibition on horses that underwent surgery for strangulating SI lesions, 38 and 23% of each group, respectively, still developed POI (74). Prokinetic drugs such as erythromycin, lidocaine, metoclopramide, or neostigmine would seem to be the logical choice in a disease process involving decreased motility, but these drugs have had controversial dosage and efficacy (69, 70, 75). The most common choice of prokinetic is lidocaine (75). A study by Salem et al. (76) evaluated the efficacy of perioperative lidocaine on 318 horses undergoing surgery for SI lesions. Perioperative lidocaine did not have a significant impact on volume or duration of gastric reflux in these horses, an integral part of the clinical definition of equine POI. The lack of universally effective pharmaceutical treatment necessitates additional case management strategies in equine patients including early feeding, supportive fluid therapy, exercise, and avoidance of formation of infection, inflammation, endotoxemia, or adhesions (69). Early feeding encourages gastrointestinal motility and sham feeding in the form of gum chewing, and placing hay within sight but out of reach has been used in human and equine patients, respectively (77, 78). As previously mentioned, mast cell stabilizers in addition to IL-1R, p38 mitogen-activated protein kinase and ICAM-1 blockers, may be potential methods to reduce the inflammatory response (52, 60, 61, 79–81). The Engel et al. (57) group that studied the involvement of memory Th1 cells suggested that future therapies may include IL-12 blockade, or other methods of preventing Th1 cell migration. Vagal nerve stimulation may also be utilized to reduce the inflammatory response and macrophage stimulation, and a laparoscopic method has been developed in pigs (82–84).

## Emerging Areas of Interest in Ileus Research

The gut microbiota has been demonstrated to change following surgical procedures, providing justification for study into its role in POI and as a novel potential target for more effective therapy (85, 86). *Bifidobacterium* and *Lactobacillus* probiotic genera have been suggested to provide an anti-inflammatory role, and potential for improving the intestinal barrier function (87–89). A 2020 study was performed in guinea pigs using *Enterococcus faecaelis, Bacillus mesentericus*, and *Clostridium butyricum* as a probiotic once daily for a week prior to initiation of POI (90). POI was initiated in guinea pigs by laparotomy with cecal manipulation. Overall, the probiotic group had significantly better motility compared to the control group, which was demonstrated by a significantly higher number and weight of feces following the procedure. Both *Bifidobacterium* and *Lactobacillus* genera were decreased after the procedure, which the authors felt as indicative of their potential role in POI. The fecal butyrate levels were also measured, as butyrate is involved in colonic transit, contractile responses, and toll like receptor stimulation (91). The fecal butyrate levels were decreased in all groups following the procedure, but the probiotic group had significantly higher levels of butyrate at 5 days post procedure. Further studies in additional animal models would be interesting to assess the potential of preoperative probiotic use in prevention of POI. While many surgeries are emergent, and do not provide a week prior for probiotic administration, recent studies in human patients undergoing gastrointestinal surgeries have shown promise that probiotic therapy initiated at surgery or up to 3 days after surgery are capable of significantly decreasing POI development (92, 93).

Within the enteric nervous system, the enteric glial cells play an increasingly appreciated direct role in regulating the intestinal barrier, and rapid induction of altered barrier function by activated glia could prove to be an important but poorly understood early contributor to POI induction. As previously discussed, it is hypothesized that tissue handling increases epithelial permeability, which allows the translocation of luminal contents, inciting and propriating an inflammatory response (43). Anup et al. (94) demonstrated in a rat model that surgical tissue handling of the SI rapidly increased conductance. Increased conductance was first noted at 30 min post manipulation, and this alteration to barrier function may be proven to occur in even shorter period of time. The inciting cause of this increased barrier function has yet to be determined. A 2020 study by Schneider et al. (95) suggested that purines like adenosine triphosphate released from tissue affected by surgical stress could activate enteric glia, which then play an important role in the inflammatory pathophysiology of POI. Glia are also reactive to mechanical stimulation, and therefore may be activated solely by tissue handling (96). Additional pathways inducing reactive glia include IL-1, S100β, glia endothelin-1, glia endothelin-B, and palmitoylethanolamide receptor (3, 97). Mice treated with anakinra, an IL-1 receptor antagonist, or antibodies against IL-1α or IL-1β had decreased POI development following intestinal manipulation (97). Activated glia produce IL-6, which is known to upregulate claudin 2, a potential method of increasing barrier permeability (97, 98). In addition, activated glia produce inflammatory products such as nitric oxide as well as pathologic levels of s-nitroglutathione, which have been shown to increase barrier permeability (96, 99, 100). If a specific pathway is identified, the prevention of glial activation may be effective in blocking the alteration in barrier function and significantly reduce the inflammatory component of POI development.

## Discussion

In conclusion, POI is a multifactorial problem with a serious impact on human and equine health. Additional study of its neurogenic and inflammatory components will be critical in further development of successful methods of treatment and prevention. A better understanding of how elements like the microbiota suppressing inflammation or enteric glial cells driving early changes in barrier permeability could highlight novel targets for more effective interventions in patients at risk for POI.

## Author Contributions

All authors listed have made a substantial, direct and intellectual contribution to the work, and approved it for publication.

## Conflict of Interest

The authors declare that the research was conducted in the absence of any commercial or financial relationships that could be construed as a potential conflict of interest.

## Publisher's Note

All claims expressed in this article are solely those of the authors and do not necessarily represent those of their affiliated organizations, or those of the publisher, the editors and the reviewers. Any product that may be evaluated in this article, or claim that may be made by its manufacturer, is not guaranteed or endorsed by the publisher.
